# Impacts and Lessons Learned of the First Three COVID-19 Waves on Cross-Border Collaboration in the Field of Emergency Medical Services and Interhospital Transports in the Euregio-Meuse-Rhine: A Qualitative Review of Expert Opinions

**DOI:** 10.3389/fpubh.2022.841013

**Published:** 2022-03-15

**Authors:** Anja Sommer, Cassandra Rehbock, Clara Vos, Christina Borgs, Sabrina Chevalier, Simone Doreleijers, Marie Gontariuk, Sofie Hennau, Eva Pilot, Hanna Schröder, Loth Van der Auwermeulen, Alexandre Ghuysen, Stefan K. Beckers, Thomas Krafft

**Affiliations:** ^1^Aachen Institute for Rescue Management and Public Safety (ARS), University Hospital RWTH Aachen, Aachen, Germany; ^2^Department of Health, Ethics and Society, Faculty of Health, Medicine and Life Sciences, Care and Public Health Research Institute (CAPHRI), Maastricht University, Maastricht, Netherlands; ^3^Public Health Department, Faculty of Medicine, University of Liege, Liège, Belgium; ^4^Center for Government and Law, Faculty of Law, Hasselt University, Hasselt, Belgium; ^5^Department of Anaesthesiology, Medical Faculty RWTH Aachen, University Hospital RWTH Aachen, Aachen, Germany; ^6^Emergency Department, Liège University Hospital Centre, Liège, Belgium

**Keywords:** cross-border, emergency medical service (EMS), interhospital transport, Euregio Meuse-Rhine, collaboration, crisis management, cooperation, EU cross-border mechanism

## Abstract

**Background:**

In the Euregio-Meuse-Rhine (EMR), cross-border collaboration is essential for resource-saving and needs-based patient care within the emergency medical service (EMS) systems and interhospital transport (IHT). However, at the onset of the novel coronavirus SARS-COV-2 (COVID-19) pandemic, differing national measures highlighted the fragmentation within the European Union (EU) in its various approaches to combating the pandemic. To assess the consequences of the pandemic in the EMR border area, the aim of this study was to analyze the effects and “lessons learned” regarding cross-border collaboration in EMS and IHT.

**Method:**

A qualitative study with 22 semi-structured interviews was carried out. Experts from across the EMR area, including the City of Aachen, the City region of Aachen, the District of Heinsberg (Germany), South Limburg (The Netherlands), and the Province of Limburg, as well as Liège (Belgium), took part. The interviews were coded and analyzed according to changes in cross-border collaboration before and during the pandemic, as well as lessons learned and recommendations.

**Results:**

Each EU member country within the EMR area, addressed the pandemic individually with national measures. Cross-border collaboration between regional actors was hardly or not at all addressed at the national level during political decision- or policymaking. Previous direct communication at the personal level was replaced by national procedures, which made regular cross-border collaboration significantly more difficult. The cross-border transfer regulations of patients with COVID-19 proved to be complex and led, among other things, to patients being transported to hospitals far outside the border region. Collaboration continues to be seen as valuable and Euregional emergency services including hospitals work well together, albeit to different degrees. The information and data exchange should, however, be more transparent to use resources more efficiently.

**Conclusion:**

Effective Euregional collaboration of emergency services is imperative for public safety in a multi-border region with strong economic, cultural, and social cross-border links. Our findings indicate that existing (pre-pandemic) structures which included regular meetings of senior managerial staff in the region and a number of thematic working groups were helpful to deal with and to compensate for the disruptions during the crisis. Regional cross-border agreements that are currently based on mutual but more or less informal arrangements need to be formalized and better promoted and recognized also at the national and EU level to increase resilience. The continuous determination of synergies and good and best practices are further approaches to support cross-border collaboration especially in preparation for future crises.

## Introduction

Crises, such as natural disasters or disease outbreaks, increase the strain on healthcare services including the emergency medical services (EMS). System preparedness, based on its readiness and adaptability, is vital to provide an adequate level of care to people in need (1). This includes the provision of high-quality care and transport of patients to the nearest and most appropriate hospital. To achieve short ambulance response times, a key factor for patient survival, inter-sectoral and cross-border collaboration are crucial (2, 3).

The novel coronavirus SARS-COV-2 (COVID-19), which reached the European Union (EU) in early 2020, had a significant and rapid impact on inter alia healthcare (1). The fragmentation of the EU in their approaches to tackling the pandemic became apparent and showed the need for public health reforms at the regional, national, and European levels (4, 5).

More specifically, the virus challenged cross-border collaboration regarding EMS. Particularly in regions such as the Euregio-Meuse-Rhine (EMR), which comprises the Southern tip of The Netherlands (South Limburg), the larger Aachen region in Germany, and North-Eastern Belgium (covering the German-speaking community and parts of the French and Flemish-speaking communities), cross-border cooperation is essential to provide appropriate patient care with the appropriate resources, including elective and emergency healthcare (6, 7). Before the COVID-19 pandemic, more than 160,000 people per year crossed the border of Belgium, The Netherlands, Germany, Luxembourg, or France to receive medical care during emergencies or elective treatments (8). Due to the EMR's characteristics, based on its geography, laws, languages, cultures, and economies, Euregional agreements, such as in healthcare, ease the collaboration between the relevant national actors. EMS and hospitals in the region are dependent on a well-functioning cross-border collaboration, though national borders remain an obstacle in the continuous development of the EMR (9–11).

With the increased spread of COVID-19, the cooperation between EU countries changed and general European benefits such as free movement {art. 3.2 Treaty on European Union (TEU) and art. 21.1 [Treaty on the functioning of the European Union (TFEU)]} were hindered if not prohibited (12–15). Cooperation can rely on general legal instruments regarding cross-border cooperation. In the European context, the Madrid Outline Convention (16) and the European grouping of territorial cooperation (EGTC) Regulation (17) are leading instruments. With respect to cross-border healthcare, the Directive 2011/24/EU of the European Parliament and of the Council of March 9, 2011 on the application of patients' rights in cross-border healthcare also serves as a basis for cross-border cooperation (art. 168.2 TFEU) (12, 18). Nonetheless, European Member States acted independently and sometimes reinstated border controls, which impacted cross-border healthcare provisions negatively (14).

**Figure 1:**
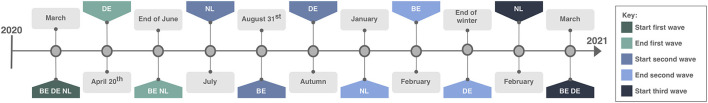
COVID-19 waves reported in Belgium, Germany, and the Netherlands (further detail on hospital surge capacity planning can be found in the Supplementary Figure 1) (19–24).

As the pandemic progressed in different waves, not every country was affected at the same time and at the same level. Figure 1 demonstrates the timeline based on the differing official dates of the first three COVID-19 waves (until March 2021) in The Netherlands, Germany, and Belgium.

This study aims to assess the impacts and consequences of the first three COVID-19 waves on cross-border collaboration between regional actors regarding prehospital care in the EMR. In doing so, we hereby distinguish between primary and secondary ambulance missions. Primary missions are emergency rescue missions including the patient transfer from the emergency scene to the next suitable hospital, where an initial stabilization and first or definite treatment can occur (25). Secondary patient transports or interhospital transports (IHT) include all patient transfers from primary healthcare facilities to a secondary or tertiary care hospital in rural areas. They are frequently necessary to provide comprehensive patient care (26), based on reasons ranging from inadequate bed capacity to a lack of a tertiary department or special equipment (27).

Investigating the impacts of and lessons learned from the pandemic on cross-border collaboration regarding EMS and IHT, we focused on: changes in cross-border collaboration before and during the pandemic, as well as lessons learned and recommendations.

This research adds a relevant and detailed analysis to some previous studies (6, 7, 9). Based on the experiences and insights of Euregional practitioners and experts in the field of EMS and IHT during the first three COVID-19 waves, this study underlines the importance of collaboration in this particular cross-border setting under these demanding circumstances.

## Methods

We conducted semi-structured interviews with experts and practitioners based in the EMR and from various fields, such as the hospital, dispatch center, and ambulance/firefighting service, to explore the impacts and consequences of the COVID-19 pandemic. The study was approved by the ethics committee of the medical faculty of RWTH Aachen, Germany (registration numbers: EK 390/20, CTC-A 20-417). All participants signed an informed consent form.

### Defining Collaboration Within the EMR Setting

For this study, we follow van Houtum's “cross-border cooperation approach” [(28), p. 73]. The focus here lies on interacting in a beneficial/useful manner based on mutual understanding. The key terms are “effectiveness, success, tools, instruments, connectivity, openness, (dis)similarities, differences, synergy, networks, cooperation, and alliances” [(28), p. 73]. The EU Directive on the application of patient's rights in cross-border healthcare similarly aims to enable cross-border healthcare safely and with high quality by promoting cooperation among the EU Member States [(3), Ch. 2, p. 35]. In the EMR, close cooperation is needed due to the shared regional challenges and opportunities defined by the geographical specifics, as well as economic and financial settings and interdependencies (10, 11). For our study, we particularly analyzed the EMR in regard to a cooperation between the several German, Dutch, and Belgian municipalities, counties, and health districts bundled together in this cross-border area. This overarching cooperation is subsequently strengthened by underlying collaborations.

We define cross-border collaboration in this study as “an activity or arrangement in the field of healthcare undertaken by two or more cooperating actors, located in different systems/countries, with the aims of transferring or exchanging patients, providers, products, services, funding, or healthcare knowledge across the border which separates them” [(3), Ch. 7, p. 219]. In the EMR, cross-border collaboration has evolved during the last decades as a long-lasting relationship among the relevant key stakeholders in public safety, and especially, in the field of EMS and IHT, collaboration is exercised as a daily routine (10, 11).

To facilitate collaboration on public safety within the EMR a group of key stakeholders [Euregio Meuse-Rhine In Geval Van Crisis/In Case of Crisis (EMRIC)] has been formed more than two decades ago. It brings together (public) organizations in firefighting, EMS, and civil protection from either side of the borders with the aim to support, coordinate, and intensify the collaboration among emergency services, health authorities, hospitals, and fire services in this border region. EMRIC is not a legal entity but organizes the Euregional cooperation of the operational structures in thematic working groups consisting of representatives of, for example, the local EMS (29, 30). From this group, the idea of the International Knowledge and Information Centre in public safety (IKIC) project was initiated and this study is a part of the project (31).

The studied region includes the Belgian provinces Liège (incl. German-speaking Community) and Limburg, the Dutch province South-Limburg, and the German city of Aachen, City Region Aachen, and District of Heinsberg in North-Rhine Westphalia (NRW). As the research area covers The Netherlands, Germany, and Belgium, the international abbreviations NL, DE, and BE are used in the results depending on the readability.

**Figure 2:**
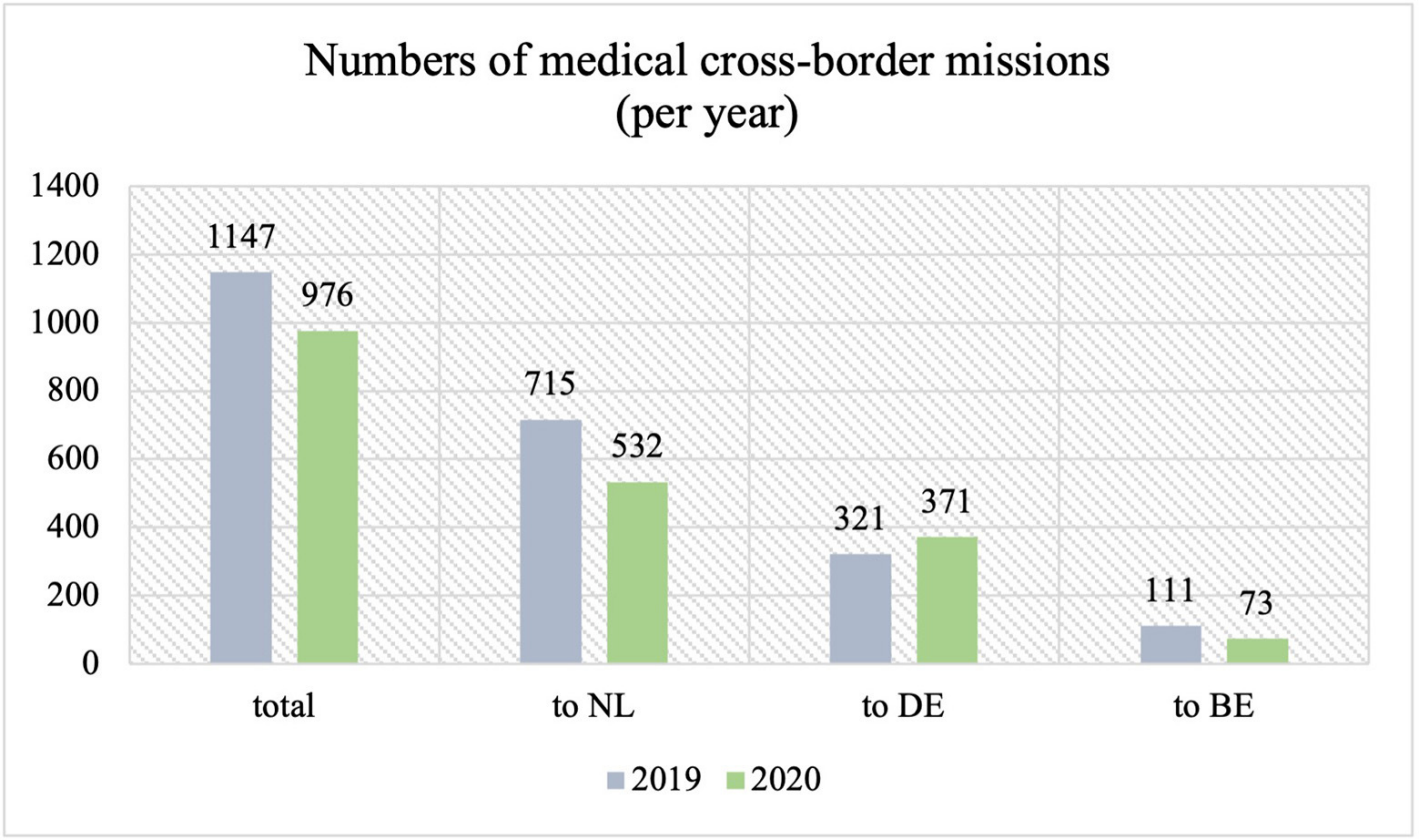
Emergency medical service (EMS) cross-border missions and interhospital transports (IHT) in 2019 and 2020 in the research area.

Figure 2 gives an overview of all medical cross-border missions in the above-mentioned research area. Overall, a total of 1,147 medical cross-border missions (EMS and IHT) were reported in 2019, of which 715 (62,3%) had the destination NL, 321 (28,0%) the destination DE, and 111 (9,7%) the destination BE. Compared to this, a total of 976 medical cross-border missions were reported in 2020, of which 532 (54,5%) to NL, 371 (38,0%) to Germany, and 73 (7,5%) to Belgium. Particularly in these cases, DE consists of the cities/regions Düren, Euskirchen, District of Heinsberg, City Region Aachen, and City of Aachen, NL of South-Limburg, and BE of Liège and Hasselt (comprising the whole EMR area). This data was provided by the Fire Department of the City of Aachen as part of EMRIC. Further data can be found in the Supplementary Material.

#### EMS Systems in the EMR

The EMS systems in Germany, the Netherlands, and Belgium, and thus also in the EMR, differ regarding their structures, operational standards and procedures, as well as their respective underlying tasks. For life-threatening emergencies, the German and Belgian emergency responses usually include emergency physician units (DE: NEF; BE: SMUR/MUG) in addition to ambulances with paramedics, whereas the Dutch system is based on paramedics only, but with considerably higher qualifications compared to the German and Belgian paramedics. Response time requirements differ, as well as the triage systems that are used by the respective emergency dispatch centers. Also, the availability of resources and the threshold (criticality) that must be reached to qualify for a medical response from the EMS system differ quite significantly, with the Dutch system having comparatively fewer resources and thus higher thresholds because of the requirements set by the Dutch government. While all these differences continue to coexist in the EMR, pragmatic and effective answers have to be found in the daily working routine when one of the dispatch centers in the region is requesting the dispatch of EMS resources across the border to allow for the fastest qualified response available to a critical emergency (2, 32–34).

### Data Collection

Overall, a total of 22 interviews were conducted according to a semi-structured interview guide (see Supplementary Material). The guide covered topics related to pre-existing cross-border agreements in the field of EMS and IHT, experiences of participants during the first three COVID-19 waves, the impact of COVID-19 measures on the Euregional collaboration, lessons learned, and recommendations for the future collaboration in the EMR. The interviews were conducted either in German, Dutch, French, or English by 5 interviewers of the study team.

Participants of the study included experts at the political and crisis management level, medical directors (EMS), dispatch center managers, physicians, as well as firefighting, and EMS practitioners from various collaborating institutions and organizations in the EMR (Table 1), allowing for a wide scope of unique viewpoints on the studied topics during the initial crisis.

**Table 1:** Details of the study participants (*N* = 22).

		**Stadt**	**StädteRegion**	**Kreis**	**Limburg, BE**	**Province**	**Zuid-Limburg, NL**
		**Aachen, DE**	**Aachen, DE**	**Heinsberg, DE**		**Liège, BE**	
Participants	Medical director EMS	1		1	1^a^		1
(function/expertise)^**^	Hospital/emergency physician	1	1		2	2	2
	Political level/crisis management^*^	1				1	1
	Ambulance/firefighting service^*^	1				2	
	Manager dispatch center				1	1	1
	Dispatch center^*^			1			

a *The participant was also a parliamentary advisor*.

* *representative*;

** *most participants fulfilled several roles but were only interviewed as for ex. manager of the dispatch center*.

Participants were invited from January to June 2021. The interviewees were actively involved in the management of the COVID-19 pandemic; therefore their availability was very limited, resulting in an extension of the recruitment period and lower participant numbers in certain regions (for example, The Netherlands) than initially anticipated.

ID numbers (ID 1-22) were used to refer to the participants' statements (Table 2). Each number represents one expert.

**Table 2:** Participant IDs per country.

**Germany (DE)**	**ID 1–7**
**The Netherlands (NL)**	**ID 8–12**
**Belgium (BE)**	**ID 13–22**

### Data Analysis

All interviews were transcribed verbatim (excluding sounds), anonymized, and translated into English by the interviewers and native speakers (except for the German interviews). Afterward, all interviews were analyzed in English or German. The software Atlas.ti Web 9 (ATLAS.ti Scientific Software Development GmbH, Berlin) was used to facilitate the collaborative coding process. This allowed joint discussions on the coding processes of each individual interview among the research group, in which the native interviewers were also partaking.

Thematic coding based on the interviews was applied (35). The coding book was carefully revised and adapted based on discussions of emerging results, allowing for a detailed overview of relevant findings. Predominant themes of the initial interview guide (see Supplementary Material) were used to structure the results according to the following sections: the collaboration before and during the pandemic, as well as the lessons learned and recommendations. To increase readability, the findings were additionally divided among the topics EMS and IHT with regard to any involved operational processes.

## Results

In total, 22 experts in the fields of EMS and IHT in the EMR were interviewed, 7 from Germany, 5 from The Netherlands, and 10 from Belgium. The experts were asked about their experiences of cross-border collaboration before the COVID-19 pandemic, changes during the pandemic, and missed opportunities in the cross-border collaboration in EMS and IHT, as well as recommendations for future collaboration within the EMR.

### Cross-Border Collaboration Before the COVID-19 Pandemic

#### Cross-Border Collaboration in General (EMS and IHT)

The cross-border collaboration in the EMR (also through EMRIC) prior to the pandemic is perceived as well-functioning and based on solid foundations (ID 1, 4, 6, 11, 16). Several respondents refer to various general agreements concerning cross-border collaboration that are in place, in addition to more specific agreements around fire protection, EMS or disaster management (ID 4, 5, 8, 9, 11). Further agreements between municipalities exist in certain border regions. These agreements take effect when one's own regional resources cannot reach a certain area in the legal time frame and can be reached faster by foreign units (ID 9). At the operational level, the overall collaboration in EMS and IHT is perceived as functional (ID 1, 6, 16).

Cross-border collaboration by means of the emergency helicopter “Christoph Europa 1” (stationed in Würselen, DE), is perceived as very well-functioning (ID 8, 18). The German helicopter conducts a lot of missions in Dutch Limburg (ID 8) and regularly supports EMS in the province of Liège (BE) (ID 18). The responsible parties of the Dutch Netwerk Acute Zorg Limburg (NAZL; Network acute care Limburg) and Christoph Europa 1 meet twice a year to exchange information and discuss any difficulties (ID 8).

While the cross-border collaboration with South-Limburg has been successful for many years and communication across the border appears to be relatively easy and direct between the services, the collaboration with Limburg North occurs infrequently and is viewed to be more complex (ID 8, 9).

There is less collaboration between BE and both NL and DE (ID 11, 18), although NL does request BE resources occasionally (ID 13). Possible reasons for less support requested by BE from the neighboring countries include better spatial coverage by their own ambulance resources close to its borders with NL and DE, differences in EMS structures, and a language barrier for the French-speaking part (ID 11). Overall, BE receives more help than it provides to DE or NL (ID 18). One expert emphasizes that in cross-border collaboration, there is never an equal balance between the countries, meaning that there is always one predominantly receiving and another one requesting resources (ID 13).

#### EMS Operational Processes

In the last years, no specific problems were reported when requesting or providing help in the cross-border setting (ID 1, 6, 16). German ambulances are usually obliged to transport patients to the nearest suitable hospital, also during a cross-border mission (ID 3). Still, in the border region, German EMS teams try to bring German patients to German hospitals and Dutch patients to Dutch hospitals, even if the hospital from the respective neighboring country is closer (ID 5, 6). One German expert emphasized that Dutch hospitals admit every patient transported by German ambulances to the emergency room (ID 3). On occasion, Dutch and Belgian ambulances also bring patients to DE if this is the nearest intensive care unit (ICU) bed available (ID 2). Cross-border care is challenging for BE as it is uncommon, and processes seem to be unknown among participants (ID 18, 19). The usual procedure in BE is to seek permission from their dispatch center to approach a hospital outside of their own region (ID 16, 18, 21). Belgian ambulances are only allowed to bring patients across the border if an emergency physician is on-scene and the foreign hospital has the specialized department required to treat the patient (ID 16, 19, 21). Between certain medical specialization departments and NL hospitals, (informal) agreements were established with the BE EMS service, including for example agreements on pediatric emergency patients, which can be transported directly to the hospital in Maastricht without requesting prior permission from the dispatch center (ID 21). One participant mentioned that the transport of German-speaking Belgians to a German hospital can be challenging because of the underlying structures (ID 18). The following illustrates a specific scenario where an exception was made regardless of the usual processes; several patients from a mass casualty event in BE (a terrorist attack on Place Saint-Lambert in Liège) were transported by Dutch ambulances to Maastricht (NL), whereas patients would usually be transported interprovincially (ID 18).

German experts feel that speaking German during a patient handover or when communicating with the Dutch dispatch center in The Netherlands is very beneficial (ID 1, 7).

“*[…] If a patient […] because he is Dutch or Belgian, or because I have the mission in The Netherlands or Belgium, want to transport him to a hospital there, I have never personally encountered problems. It always worked great to place the patient there, yes, one does not exactly know who needs to be called, because that has changed several times especially in Maastricht, now. But still you were always welcomed in a friendly manner and from my point of view it works much more smoothly than here in Germany” (ID 1)*.

Concerning the acceptance and recognition of educational background and competencies, each country accepts those of the foreign EMS staff, and everyone works according to their own standards and standard operating procedures (SOPs) for treatment (ID 5, 11, 20). Related to this, two BE experts are under the impression that the different levels in acute care training and the differences in performing medical procedures by the various urgent care teams are problematic (ID 14, 15). One Belgian expert also describes the differences among the EMS vehicles as a challenge, as regular emergency physician vehicles do not exist in NL, neither the Paramedical Intervention Team (PIT) (higher qualified staff compared to regular ambulances in BE) in DE and NL, and because Belgian ambulances are not legally recognized in DE (ID 17).

In general, handover procedures are carried out according to the guidelines of the country to which the patient was transported (ID 20). In the District of Heinsberg, a cross-border handover protocol (DE and NL) is in place, which was developed by an EMRIC working group and helps to give a structured overview about the most important handover facts in Dutch and German:

“*[…] The EMS expects how to hand over the patient now, as quickly as possible, as it is in Germany. And in The Netherlands they have such expectations, what's next, and do they announce it to me correctly, do they make a sensible handover for me?! And I think this structured protocol has contributed to this […] because it is then relatively clear, okay, they will want to know that from me soon. And so that you have it available, that's why it's [stored] on the vehicles, then we take […] and fill it out or browse through it when it comes to the registration” (ID 6)*.

In EMS, the pre-announcement en-route to the receiving hospital is done by calling the destination hospital directly (independently of where the hospital is located) (ID 1, 9, 10, 21):

“*So I try to call the hospital directly with the numbers I have, so that it doesn't have to go through the dispatch center from Aachen to Maastricht from Maastricht to the hospital' I'm actually lucky that someone always speaks German […]” (ID 1)*.

This facilitates that the emergency department staff is adequately prepared for the arrival of a new patient; pre-announcement is especially expected from Dutch hospital staff (ID 10).

#### IHT Operational Processes

Cross-border IHT between NL and DE are regularly performed, while everyone concentrates on their own processes, no difficulties are perceived (ID 1, 2, 7):

“*The Dutch hardly ever transport interhospital to Germany. They have a functioning, adequate health system with a […] much better-appearing organizational structure in terms of intensive care transport from hospital to hospital […]. Belgium also has a well-functioning health system, which is of course oriented towards Liège and Brussels. […] Our contact region is East Belgium with the German-speaking area, which by law is fundamentally entitled […] to be treated by someone who speaks German” (ID 2)*.

Cross-border IHT from BE to DE or NL is possible (ID 16, 20), although the transport between hospitals is not considered to be emergency medical care in Belgium but is rather classified as non-urgent patient transport (ID 20) and is usually only coordinated within certain provincial networks nearby (ID 18). In addition, cross-border IHT from Belgium are only allowed in certain circumstances under certain conditions (ID 15, 18) and the BE system favors transferring patients within the same province or to the next Belgian province (ID 18). If a child in critical condition needs intensive care and nearby Belgian hospitals with a pediatric ICU are not available, one expert reports very positive experiences in referring children to the hospital in Maastricht (ID 19). For pediatrics and neonatology, certain agreements exist between hospitals in BE and NL (ID 21). This is in line with the (informal) agreements for EMS to transport children directly to Maastricht (ID 21). The expert was impressed by the level of care *via* the mobile intensive care unit (MICU) retrieval teams from Maastricht as shown in the past (ID 19).

Requests for patient takeovers and IHT from NL or BE to DE are particularly very rare (ID 2):

“*Interhospital transfers from The Netherlands [are] an absolute exception. And if that's 3 in the year, it's a lot” (ID 2)*.

Nonetheless, a strong collaboration between the hospitals in Aachen and Maastricht exists (ID 8). When performed, cross-border IHT generally operate the same way as within their own country; a transport request is made and if a bed is available at a certain hospital, the IHT will take place (ID 9, 11). In addition, IHT between the hospitals in Eupen (BE) and Aachen take place and are organized informally, without involving the dispatch centers (ID 13).

A collaboration between the MICU in Maastricht and the MICU in the Region of Aachen is in place but is rarely used (ID 1). IHT in NL provided by a German MICU usually does not happen (ID 3). Only if the Dutch MICU is not available or does not have the capacity to perform a certain IHT, a German ambulance or MICU is requested to pick up the patient in The Netherlands or the other way around (ID 3, 5).

#### Operational Dispatching Processes (EMS and IHT)

Communication among dispatch centers in the EMR is unproblematic and straightforward according to one NL expert (ID 8). The responsibility for EMS missions lies with the country where the incident happened, however, if a cellphone call is wrongly directed to one dispatch center due to the proximity to the border, the call needs to be referred to the responsible dispatch center (ID 5):

“*[…] a classic example would be that in Vaals […] there is an emergency, and the Dutch then contact us to ask whether the ambulance station 7 [in Aachen] is manned by an ambulance and if that is the case, it will then be dispatched to Vaals, […] but remains a mission in the responsibility of the Dutch. […] If that is an emergency request by mobile phone from Vaals, it then arrives at the dispatch center [in Aachen] and they now see ok, we also have the ambulance 7 available, then, of course, they could say, we will send it to you directly because that is also actually possible according to our German regulations [..]. But we are not allowed […] we forward this emergency call to Maastricht, […] they make the decision whether they want the corresponding support from Aachen or not” (ID 5)*.

Similarly, for IHT cases, the responsibility lies with the dispatch center in the region where the patient is picked up (ID 5). Consequently, patients can be transferred over the border without the need to inform all involved parties (ID 5). For example, a transport provided by NL from a Dutch to a German hospital only involves contact and information exchange between the Dutch hospital, the Dutch dispatch center, and the German hospital. The German dispatch center is not informed or involved, and the contact between hospitals is usually by telephone (ID 6). Generally, in the region of Heinsberg, it is perceived that the Dutch dispatch center calls more often for help from the German dispatch center than vice versa. However, a special vehicle for infant transportation is sometimes requested from the dispatch center in Maastricht as it is the closest special-purpose vehicle in the area around Heinsberg and Aachen (ID 7).

In NL, the regulations and guidelines for the quality parameters of EMS systems are described to be different compared to DE regulations. One example is that the required time to arrive at the scene is monitored much stricter in NL and can influence their decision-making to request help from neighboring EMS:

“*[…] for the Dutch, it is very important how for example the time of arrival is and when the German vehicles [arrive]. So […] they alert a German ambulance, then they have to be able to understand exactly how long this ambulance has now required [to arrive at the scene]” (ID 5)*.

To reach their target of a 15-min arrival time to very critical patients (urgency level A1 in NL), the dispatch center in Maastricht regularly requests ambulances from DE due to their closer proximity to certain areas such as Vaals (NL) (ID 11). The Dutch EMS can only fulfill the time limit of 15 min in more than 95% of the A1 (highest urgency) rides with support from the German EMS (ID 11). On the contrary, the Dutch EMS are well-placed to cover the border-region to Belgium with their own resources and consequently require less support from Belgium. In BE, DE or NL ambulances may only be requested if they can reach the emergency scene at least 10 min before their own BE resources (ID 13).

When it comes to a request for help, the Dutch dispatch center, for example, calls the German dispatch center to share the most important information and the relevant emergency keyword. However, the German dispatcher decides which emergency keyword will be chosen to alert the German EMS resources. The decision to dispatch a certain urgency level or EMS response may differ from the original request because the Dutch and German emergency keywords and processes are different (ID 7):

“*[…] I have a heart attack on this and that address […][it is] the same as what we ask. But in the end, we have to think about it ourselves, does that fit our […] emergency keyword or not? If we now have a heart attack, [we] will just send an ambulance plus physician there, regardless of whether they [originally only asked] for an ambulance” (ID 7)*.

### Cross-Border Collaboration During the COVID-19 Pandemic

At the beginning of the pandemic, the collaboration proceeded at the same level for non-COVID-19 patients. On the contrary, for patients with COVID-19, cross-border collaboration was only possible *via* personal and informal agreements between stakeholders. Over the course of the pandemic, cross-border missions and transfers decreased in cases of non-COVID-19 patients and patients with COVID-19 were only transferred across the border after introducing official coordination measures at the national level (ID 2, 3, 4, 5, 8, 9, 10, 12, 13, 17, 20).

Respondents consider the collaboration between NL and DE before and during the pandemic to be particularly good since it is based on already existing partnerships (ID 5, 8, 11).

#### Cross-Border EMS Missions During the Pandemic (Non-COVID-19 Patients)

Some experts assumed that cross-border missions still took place in the same quantity as before the pandemic (ID 1, 10), while others felt that cross-border interaction decreased especially with Belgium (ID 3, 16). Decreasing numbers of cross-border cases between Aachen and BE and between NL and BE have also been experienced before COVID-19 (ID 3, 11). After the initial phase of the pandemic, the number of cross-border missions returned to the same level between Heinsberg and NL (ID 6).

#### Cross-Border IHT Missions During the Pandemic (COVID-19 Patients) Before the Implementation of National Planning Tools

Because Germany has more ICU capacity than Belgium and The Netherlands, requests for taking over patients with COVID-19 over the course of the pandemic were mainly directed toward Germany (ID 13, 10, 21).

Cross-border IHT collaboration between NL and DE was more common in the first peak of the pandemic when insufficient ICU beds were available in Limburg (NL) and patients were transferred to Germany (ID 8, 9); this was based on the existing relationships between Maastricht, Aachen, and Venlo, but also between Enschede, Twente (NL), and the corresponding German hospitals (ID 8). The communication and initiation of this IHT happened at the hospital level (ID 9).

The number of patients who were transferred to German hospitals decreased as the pandemic proceeded. Patients were mainly transported to other Dutch hospitals, especially when regions were labeled as high incidence areas (ID 4).

“*So the Dutch first and foremost allocate within the country before they go abroad, which is actually not the normal situation. The normal situation near the border is actually […] that one also exchanges across borders. Amidst high incidence, that doesn't happen currently” (ID 4)*.

For BE, cross-border IHTs were stopped and BE did not accept foreign patients during the first two waves. Anything that fell within the EMS agreements (outside of COVID-19) continued and the general rules were followed. Patients with COVID-19 were not transported abroad, and neither were any accepted at the beginning (ID 20), except for specific cases (ID 13, 21), which were transported abroad by the Belgian military (ID 21).

During the second wave and only if national resources were not available, BE patients were brought across the border, outside of the Belgian hospital network (ID 13, 20). The province of Liège was impacted significantly by the pandemic and around 230 patients had to be transferred (which accounts for their entire provincial ICU capacity) (ID 13, 17, 18). Of these 230 patients, 30 patients were transferred to DE (ID 13). Also, patients could be transported, who under normal circumstances would not have been allowed to be transferred due to the national guidelines (ID 14). Normal cross-border EMS cases, excluding COVID-19 cases, continued during the pandemic as regulated by the official national agreement between NL and BE (ID 20). In addition, extraordinary agreements for patients with COVID-19 were implemented at the national level which led to the take-over of Dutch and French patients in BE Limburg and the transfer of Belgian patients to Germany. These IHT did not create any obstacles (ID 20), even though there were a few concerns and cross-border IHT were closely monitored by a sub-team in BE that organized the cross-border transfers (ID 13). In rare cases, German ambulances or MICU picked BE patients up in Belgium and transferred them to Germany. German ambulances often also transferred Belgian patients back to BE; this was also the case for Belgium, which sent out resources to pick up patients in Germany and transferred them back home (ID 13). This allowed for flexibility in cross-border IHT and created more resource capacity in BE (also for non-emergency transports) (ID 13). Patients were only transferred back to Belgium if they were no longer in need of ICU treatment and received their final treatment in BE hospitals (ID 18). Other patients, however, completed their hospitalization in the foreign hospitals across the border and at times deceased there (ID 18). One Belgian expert explained that BE hospitals selected which patients were viable for cross-border transport very carefully. This seemed to be well-appreciated by receiving hospitals since patients did not arrive in unstable critical conditions (ID 18).

#### EMS and IHT Operational Processes

With the onset of the pandemic, Belgian hospitals (mainly Verviers or St. Vith) directly asked the university hospital in Aachen to take over ICU patients; this was coordinated as per usual *via* telephone between the physicians (ID 2, 16). One expert stated that the tele-EMS physician in Aachen played an essential role in pre-announcing cross-border ICU patients at the respective hospitals for IHT (ID 4).

At a political level, Germany agreed to take over BE patients when BE reached its capacity limit (ID 16). When certain missions were officially initiated at a political level, cross-border IHT with national resources took place but Belgian patients were also transported by German ambulances while billing issues remained unclear (ID 4). One expert explained how help is always provided first, and legal questions need to be discussed afterward (ID 9):

“*If another dispatch center calls for support, we will arrange the support and whether or not this is allowed, we will see afterward. […] it's all about the patient's health in the first place and the rest will follow” (ID 9)*.

The process of IHT between the hospitals in Eupen and Aachen changed during the pandemic and was organized formally *via* the dispatch center in Liège (ID 13).

Organizing transfers from BE to DE was very challenging and time-consuming and the coordinating team had to be able to speak French/German or French/English (ID 13). Already existing contacts through EMRIC and the medical working group cross-border emergency medical assistance in the EMR (EUMED) facilitated the transfer of BE patients to the close border region around Aachen in Germany (ID 13).

IHT processes included the ongoing contact among the DE and NL EMS and the director of the trauma center in Maastricht. Neither side considered it necessary to change previous habits and operational processes, including differences in personal protective equipment (PPE) or hygienic measures (ID 3, 9, 11). National, regional, and local standards and measures were recognized by the other countries just as before the pandemic (ID 9, 11).

“*No, we actually asked [the responsible persons from the Dutch EMS and from the Maastricht University Hospital] […] whether we come into conflict with our somewhat different PPE concept […] and they said: 'You do as you want, and we do as we want. And we also accept your scheme as you want, so everything is not a problem'” (ID 3)*.

The national treatment guidelines of patient care in the cross-border setting could still be applied by the EMS teams as before the pandemic:

“*Overall, as far as I know, the cross-border collaboration continued to function just as it did before. And the Dutch came over to us for missions and […] treated a COVID-19 patient according to the Dutch system and we did the same […] in The Netherlands or Belgium” (ID 5)*.

#### Resource Capacity and Management During the Pandemic

Resource requests from the dispatch center in Maastricht to the district of Heinsberg (which was hit seriously during the onset of the pandemic) decreased significantly. The dispatch center in Heinsberg was also not always able to provide help when asked for resources, as its own resources were very scarce (ID 7).

Capacity problems in the initial phase of the pandemic in hospitals on both sides (NL and DE) led to hardly any patients being accepted from across the border. For example, only Dutch patients or patients living in the border region could be brought to a Dutch hospital, but otherwise, foreign patients were not accepted in NL (ID 7) and neither in BE (ID 20). German patients from Heinsberg were therefore often not transported to Dutch hospitals when resources were scarce on both sides of the border (ID 7):

“*And then they said, […]'we're full, right, go to your hospitals'; we hardly asked then because it was difficult to explain to the German there that he was going to be driven to Holland, where the incidences are perhaps even higher. […] then, of course, they didn't really want that either” (ID 7)*.

Admitting patients with COVID-19 to an ICU implies long-term hospitalization and ICU treatment. Hence, tying up considerable resources for a long time for one patient needs to be considered when agreeing to accept foreign patients (ID 2, 18).

#### Information Exchange During the Pandemic

A Euregional dashboard facilitated the exchange of essential information (ID 4, 14). One main challenge, however, was the different approaches to processing certain information, like differing working and decision-making processes based on different factors. For example, in Germany, decision-making was based on incidence numbers (ID 4).

Crisis management team discussions in the City and City Region of Aachen included data of EMS resources and bed capacity planning of hospitals in the EMR (including COVID-19 patient numbers and bed capacities, which are updated daily on the NL and DE side) (ID 1, 11). This data was also shared with BE (ID 14). Other than that, cross-border cooperation was not discussed any further (ID 1, 11). Additionally, another expert from a different region was not aware of cross-border topics being discussed in the crisis management teams (ID 10).

Close communication between actors across the borders (federal police Germany, EMR, EMRIC, other crisis management teams) remained intact (ID 4, 5, 20), but no contact person from NL or BE was actively involved in the local crisis management teams in Germany (ID 4,5).

Experts working operationally experienced that at a political level, cross-border support was promised very quickly and seen as having worked very well-despite the fact that detailed information of cross-border rescue missions (who brings a patient from Belgium to Germany for example) was not properly exchanged and the execution was left to the operational EMS teams (ID 1, 2). Additionally, it was difficult to give definite numbers to foreign countries on how many patients could be received, as hospitals had to make sure they were available for patients in their own region (ID 2).

“*[…] There was this appeal from Belgium to take on patients. […] so it happened one evening that a patient was supposed to be transferred from Eupen, who was completely stable […] and for whom a primary physician vehicle had to [be dispatched] […] and that was [a] totally useless waste of resources and then you only get told from the upper level that this is a highly political matter, we have to do that now, and I just think that's wrong, […] if you already have a crisis management team, and if you have such a big situation, then […] you can't personally boast about: 'I always have an emergency physician for you […]' […] But thank God that only happened once” (ID 1)*.

“*In the end, you need a setting where it is clear [that] when […] two governments at federal or state level say that this is now [the] concept: Patients are transferred from country A to country B - […] it cannot be that the discussion starts [again] via the micro-management [level] with the respective manager or dispatcher from the EMS […], about which vehicle is going where and whether it can drive with a special signal and how many people… can join there and if a patient is [transported], who will pick him up and whether the Belgian EMS will have to do that […]and who informs him” (ID 2)*.

One expert, at a higher managerial level in the EMS, had an opposing viewpoint and could not report any difficulty at the operational level (ID 5):

“*So in the […] operational business […] nothing became known [to me] that there were difficulties […] somewhere” (ID 5)*.

This was supported by one German expert at the crisis management level who concluded that much more intensive cross-border communication and exchange took place (ID 4). Also, new Euregional working groups were created within EMRIC, forming a new European project named PANDEMRIC (ID 4).

No problems were reported to the experts regarding border closings; these had no operational impact for EMS or IHT (ID 5, 7, 8, 9, 16, 17, 20). One expert explained that the fear of border controls led to an arrangement where BE ambulances were being escorted by police forces to avoid any transfer problems at the borders (ID 14).

#### The Implementation of New National Planning Tools During the Pandemic (COVID-19 Patients)

All three countries eventually implemented changes to the national organization of IHT, especially for patients with COVID-19 during the pandemic.

In NL, IHT had to be managed according to national guidelines and through the regional [Regionaal Coördinatiecentrum Patiënten Spreiding (RCPS)] and national coordination center [Landelijk Coördinatiecentrum Patiënten Spreiding (LCPS)] for both the national and international IHT. This was a major obstacle to the cross-border collaboration between NL and DE as this made Euregional or direct communication and transfers impossible (ID 5, 8, 10, 11).

“*So the only thing I found unfortunate, especially when the LCPS really came onto the scene, that they were very much attached to the national borders” (ID 10)*.

International transports in Belgium were organized by the central government, for which a national task force was established: the Surge Capacity and Transport Taskforce (ID 17, 20). In contrast to before, these transports were now organized *via* the dispatch center in Liège (ID 17). One expert criticized information from the task force as being insufficient, as hospitals and EMS were not informed properly about capacity in other regions (also across the border within the EMR) (ID 21).

A new coordination point in Münster (Germany) was introduced focusing specifically on the coordination of cross-border IHT in Germany (ID 2, 9). This center is responsible for the allocation of ICU resources. Some experts explained that the center did not coordinate cross-border IHT but was rather a point of contact (ID 2).

“*The state government and the federal government provided regulation for Covid treatment within the framework of this cluster regulation, […] a communication center […] attached to the university hospital in Münster, which primarily […] should take over the locating of intensive care resources. […] That was well thought out on paper. In reality, it was the case that the staff in Münster took calls and then said […] ‘call the nearest hospital and talk to them' […], then we had to refer them to Münster and then the hospital in Münster said to the dispatch center ‘yes, but you have already called Aachen, then call again'. And when we said ‘we had no capacity', he would call Münster again and say ‘Aachen says they have no capacity', and then maybe the colleagues in Münster suggested a second hospital, where he then had to call as well” (ID 2)*.

The coordination point redirected any call for help to, for example, the Western Single Point of Contact (SPOC) center (one of the five national coordination points for IHT during the pandemic in Germany) (ID 4, 5). Dutch and Belgian staff were not properly informed about the coordination center in Münster (ID 2). Nonetheless, experts reported a few IHT (NL to DE), which were organized *via* the coordination point (ID 3).

One of the Dutch experts explained that a lot of distress was caused for Dutch families when patients were transported to Germany organized *via* the coordination points LCPS and Münster. The LCPS does not consider the “human-factor” but simply chooses the next hospital from a list of available locations provided by Münster, even if it is very far away (ID 10, 11, 13). This leads to dramatic consequences for patients and their families because of large distances and, e.g., families needing to stay in holiday apartments to be close to their relatives (ID 11).

“*[…] there [was] an unbelievable amount of misery […] in bringing the patients to Germany. That had an impact on the families” (ID 11)*.

In cases where Dutch patients were transported far outside the border region, every family was supported by a Dutch social worker and contact person (ID 11). Additionally, the coordination at the national level caused IHT from NL and BE to German hospitals outside the border region, which are not used to receiving patients from other countries (ID 13). Some problems occurred such as foreign helicopters landing in the wrong location (ID 13).

### Possible Lessons Learned and Recommendations Regarding Cross-Border Collaboration in the EMR

As emergency situations or dangers do not stop at borders, cross-border collaboration is seen as very important (ID 4, 8, 13, 22):

“*I find the cross-border collaboration extremely exciting. […] I think it is right and important to continue working on this topic and such a situation, where there is certainly danger inherent, must never lead us to fall back into situations where there is no longer any cross-border collaboration because also the danger […] does not stop at the borders” (ID 4)*.

“*I think it's very important to have the cross-border collaboration because our region has so many borderlines connected to Germany and Belgium that is for us it is more or less a partner of which you think very often where you could collaborate perhaps” (ID 8)*.

Especially when it comes to determining which ambulance could be fastest at the emergency scene or which hospital is nearest, borders and differences in billing (or other) should not play any role (ID 22):

“*I think our health insurance differs on a number of points, but when it comes to emergency medical care, I don't think that should be a factor. Then it really comes down to human lives and patient care. I think it should take precedence over the rules and other interests” (ID 22)*.

More central structures for communication and crisis management plans should be developed for cross-border collaboration in catastrophes and disease outbreaks, such as COVID-19 (ID 2). Further positive aspects related to communication, preparedness, general collaboration, and processes of cross-border collaboration in the EMR are summarized in Table 3.

**Table 3:** Positive aspects of the cross-border collaboration in the Euregio Meuse-Rhine (EMR).

**Area**	**Positive aspect**	**Participants (ID)**
Communication	Structured and regular communication is very helpful, especially with EMRIC and the Euregio Meuse-Rhine as an organization	4
Communication	Communication generally functions well across the borders as it is based on a strong network	9
Preparedness	Exercises and preparation within EMRIC are useful for certain scenarios in cross-border collaboration	8
General collaboration	Good collaboration with EMRIC	16
General collaboration	Good collaboration between Eupen (German speaking community in BE) and Aachen (DE) for EMS	16
General collaboration	Considerably less collaboration between the hospitals in Maastricht and Eupen, but still works well	16
General collaboration	Positive cross-border collaboration between hospitals: on operational level in the ICU etc. very good and easy collaboration but on organizational level very complicated and difficult	2
Processes	Processes between South Limburg (NL) and Germany are improving more and more; advanced notification of German EMS to Dutch hospitals works well	10
Best practice exchange	Monitoring and exchange about national COVID-19 measures was helpful to learn from each other and allowed for aligning/adapting hygienic measures, such as wearing masks (especially in the beginning of the pandemic)	18

Additionally, the following challenges and lessons learned from the first three waves of the pandemic were described by the experts and should be addressed in the future.

#### Challenges in General Collaboration

The region still lacks an exchange of best practices and experts highlighted the importance of finding synergies between the different systems. Very significant differences continue to exist between the three healthcare systems. However, the different systems could learn more from each other, especially as they are within such close geographical proximity of one another (ID 1, 2, 6, 22).

“*[…] So from my point of view there is enormous potential for cross-border collaboration, yes, or… also hospital organization, triage system or emergency room in Maastricht, and also in Heerlen, […], well I have never experienced that when I was there,[that] there was chaos. In Germany, however, there is regular chaos, that is what is going wrong with us, or differently, […]” (ID 2)*.

As one specific example, the exemplary management of methicillin-resistant *Staphylococcus aureus* (MRSA) infections in NL can be named. This, however, poses a challenge in the case of IHT (except for ICU patients) where a patient is brought to a Dutch hospital (be it from DE or BE) because a lot of administrative steps are involved. Patients will be isolated in the emergency department at the beginning of their hospital stay posing a barrier for cross-border care (ID 21).

Regular cross-border meetings stopped during the pandemic because countries mostly focused on their own systems (ID 5, 21); therefore, it was also assumed that if the neighboring country needed support, they would have requested it (ID 5). Every region focused on its own situation instead of keeping up regular exchanges across the border and learning from each other (ID 5).

“*This regular exchange has fallen asleep. […] So ‘a great idea, we didn't even have it yet', or ‘a cool idea, we'd like to implement that too'. That has not happened in this context because we were all very busy at first […] and then we say yes, as long as I don't hear anything from the other […], they are probably still fine […] To continue [the] existing structures and to continue the exchange and just not have such a cut […] Well, I think nothing went worse [by not having] this contact, but it might have gone better if we would have had contact” (ID 5)*.

The next step for the EMR should be to address how the three countries can support each other's public health system:

“*I think we need to improve our treaties. […] when it comes to the Euregion […] for what we are going to do in these kinds of situations. So we've arranged our emergency medical service well, but that's assuming it's about individual patients, and small groups of patients […]. […] Not in such an overwhelming pandemic really, where the entire public health system is compromised. And the next phase that we need to do is make plans together – with each other, I mean in this region – how we can support the structure of public health of each other, without compromising the functioning. […] […] how can we […] all make sure within Europe that we can manage that, without having to undermine our normal structure” (ID 20)*.

Currently, this expert does not see a solution for this in short term but sees the need for a European healthcare system in the future (ID 20). At the European level, the allocation of patients should be improved by centralization of expertise where patient groups with low numbers but high variability can be treated (ID 20).

However, various barriers were identified to limit the potential of cross-border care, especially in Belgium (ID 19), such as cross-border collaboration within national policy-making:

“*[…] Look, national thinking is just a hindrance, isn't it? If you just see how Maastricht, how close that is to us if you would start working together with it and open and involve things, but, yes… I think that's a utopia. […] We have to focus on the governments after all […] so that [initiative] has to come from above” (ID 19)*.

One specific example where differences in legislation led to operational problems during EMS missions is the transport of a detained psychiatric patient based on a psychiatric medical report for compulsory hospitalization since it is not as straightforward when bringing the patient across the border (ID 1).

“*[…] the transfer of a detained psychiatric patient across the border. There I have the opinion, that that transport can be carried out [and finished] according to the state law, where the transport started. The Dutch think [that] we have to meet at the border crossing point. […] that's the only thing I know where it doesn't work smoothly” (ID 1)*.

Some experts had little knowledge about cross-border collaboration and were not aware of the impacts and consequences of the pandemic on the collaboration (for example Euregional meetings, information exchange, and consideration at the national level) (ID 10, 11, 15, 16, 18, 21, 22). Some experts were also not aware of any cross-border agreements before and during the pandemic (ID 10, 13, 16, 22) [e.g., reimbursement (ID 17)]. A summary of the findings is gathered in Table 4.

**Table 4:** Examples of lessons learned and recommendations for general collaboration (based on encountered challenges before and during the first three COVID-19 waves).

**Area**	**Lessons learnt**	**Recommendations**	**Participants (ID)**
General	Recommendation	Best practice exchange and synergies between systems	1, 2, 6, 22
collaboration	Recommendation	Keeping up regular exchange and learning from each other	5, 21
	Recommendation	Supporting each other's public health system: no solution yet but believe in a European healthcare system and allocation of patients at European level	20
	Recommendation	Inclusion of cross-border care in national policy making	19
	Lesson learnt	Complex administrative steps in case of IHT from BE/DE to NL (because of MRSA measures)	21
	Lesson learnt	Lack of expert cross-border knowledge and related impacts of the pandemic	10, 11, 16, 21, 22
	Lesson learnt	Time and resources are lacking to sustain collaboration in general	8
	Lesson learnt	Uncertainty regarding contact points results in less collaboration between DE and BE compared to DE and NL	1
	Lesson learnt	NL and DE collaborate more because of geographical reasons	9
	Lesson learnt	Collaboration with BE is generally less and stopped mostly during pandemic	3, 8, 21
	Recommendation	Regular cross-border meetings between operational staff (not just at political level)	7
	Recommendation	Language course in medical Dutch for German dispatchers	7
	Lesson learnt	Crisis management teams did not discuss cross-border patient care, IHT nor capacities but focused on citizen-related impacts (incl. border closing)	6
	Lesson learnt	No patients from Belgium were transported to Aachen (DE) anymore as the pandemic progressed	2
	Recommendation	Knowledge about the other systems, staff education, and competences and increase information about legal coverage of operating in other countries	15
	Lesson learnt	Cross-border transports involving detained psychiatric patients are not working smoothly	1

#### Agreements

While a cross-border agreement on emergency medicine between NL and BE has existed for over 10 years (ID 13), an overall cross-border agreement between Belgium and Germany at the national level including the health insurance companies is missing. Consequently, the exact details of emergency coverage remain unclear (ID 2, 16, 17, 19, 20). One BE expert explains that administrative processes must be checked case by case after, for example, an emergency transportation (ID 20). An agreement between BE and Rheinland-Pfalz (another state in DE) does exist, but negotiations over an agreement between BE and NRW have been ongoing since 2010 (ID 13, 17). Generally, the Belgian EMS system prefers the treatment of patients in Belgian hospitals over other hospitals in the EMR (in DE and NL). Between BE and DE, there is currently no standardized way of communication to handle cross-border emergency transports nor planned IHT (ID 2, 17, 19), partly because it does not seem too relevant for the daily work of EMS in the region (ID 2, 19).

One expert reported that even though certain agreements exist, rumors still emerged in the past that cross-border care had to be paid for by the patient, which is incorrect (ID 19). The expert also saw unclear billing issues as a major challenge as well as uncertainties around the topics of working processes, education of staff, patient transportation, and responsibilities of physicians for patient care (ID 19). The expert further stated that generally, mostly informal agreements and processes exist but hesitancy remains as to whether all the information is available to the expert (ID 19). Information exchange regarding the agreements and processes could be significantly improved (ID 19).

Another expert perceived the existing Euregional agreements as non-applicable during the pandemic and suggests improving those (ID 21). A summary of the findings is gathered in Table 5.

**Table 5:** Examples of lessons learned and recommendations concerning formal Euregional agreements (based on encountered challenges before and during the first three COVID-19 waves).

**Area**	**Lessons learnt**	**Recommendations**	**Participants (ID)**
Formal Euregional	Recommendation	Harmonization of legislation in the EMR, starting with harmonizing operational processes in BE	17
agreements	Lesson learnt	Existing agreement between NL and BE	2, 16, 19, 20
	Lesson learnt	Agreement lacking between DE and BE: uncertainty with health insurance coverage and financial aspects; Case-by-case assessment required	2, 16, 19, 20; 20
	Lesson learnt	Treatment at national level is preferred over cross-border care by BE (EMS and IHT)	2, 19
	Lesson learnt	BE and DE lack standardized communication processes for cross border EMS and IHT missions; partly based on lack of relevance in their daily work	2, 19
	Lesson learnt	Lack of awareness of agreements	10, 13, 16, 22
	Lesson learnt	Lack of awareness also leads to (untrue) rumors	19
	Lesson learnt	Unclear billing and working processes, staff education, patient transportation, CO2 standards of vehicles and responsibilities related to cross-border patient care (especially for BE)	3, 14, 19
	Lesson learnt	Informal agreements in general exist but uncertainty on level of available information	19
	Recommendation	Information exchange could be improved; the care and quality for that exists (for example *via* an international or European platform)	17, 19

#### Process Changes During the Pandemic

Although cross-border collaboration worked well during the pandemic, emergency and disaster management plans including cross-border care should be developed and agreed upon before such a situation becomes urgent (ID 14, 17):

“*In terms of collaboration […] If I sum up, it went very well, honestly, everyone showed a lot of goodwill for me, [but] it should be written in advance in texts and not when it's the moment to say to each other, but what do we do now?” (ID 14)*.

In addition, these plans should be tested in large-scale simulations including cross-border collaboration in such pandemic situations (ID 17).

Further, agreements and regulations on cooperation should ideally be the same between all countries (DE-NL, NL-BE, BE-DE) (ID 15).

Material and/or equipment was shared scarcely between different countries in the pandemic because:

“*Every country in Europe has had shortages. […] Every country has reacted very paternalistically. For example, the large stock of masks […] Germany and both France […] banned the export of masks, which has put other countries in trouble. And nobody was prepared, had such a big stock around” (ID 20)*.

Support was not provided to other European countries that were struggling from capacity bottlenecks at certain moments in time, while resources and beds in the EMR were still available (ID 22).

“*That European solidarity has had practically no role” (ID 22)*.

A summary of the findings is gathered in Table 6.

**Table 6:** Examples of lessons learned and recommendations about process changes (based on encountered challenges during the first three COVID-19 waves).

**Area**	**Lessons learnt**	**Recommendations**	**Participants (ID)**
Process changes	Lesson learnt	National IHT processes in South Limburg (NL) interfered with regular cross-border agreements (for ex. choosing the closest hospital)	10
	Recommendation	Definition of explicit criteria to transport	12
	Lesson learnt	Little exchange of material or equipment in Europe; Lack of European solidarity (some areas were more affected than others)	20
	Lesson learnt	Procedures and standards for IHT in pandemic situations	13
	Recommendation	The EU could impose standardized crisis management for EU countries	13

#### Knowledge About Availability of Cross-Border Resources

National bed capacity tools could be improved by including live/current bed capacity (including those of ICUs and special hospital departments) from across the borders as well (ID 1, 2, 7, 8, 17, 21) because resources in the EMR are not adequately used at the moment (ID 1, 8). These systems should be compatible with each other and allow information exchange to improve collaboration (ID 21). One central coordination point for IHT could be implemented (ID 2).

Regarding the availability of resources and specialized treatments/departments, it seems difficult to know for the German EMS workers which hospital in NL can offer which treatment at which time (e.g., cardiac catheter examination), as the patient intake rotates between hospitals in the NL region (ID 1). A summary of findings is gathered in Table 7.

**Table 7:** Examples of lessons learned and recommendations on the availability of cross-border resources (based on encountered challenges before and during the first three COVID-19 waves).

**Area**	**Lessons learnt**	**Recommendations**	**Participants (ID)**
Availability of cross-border resources	Recommendation	High need for transparency: Implementing live bed capacity numbers in national bed capacity tools, including the ICU and across borders (online cross-border data registration system)	1, 2, 8, 12, 13, 21
	Recommendation	Real-time data gathering in the coordination centers LCPS	12
	Lesson learnt	Inadequacy of resource usage in EMR	1, 8
	Recommendation	Improving compatibility among national registration systems and information exchange	21
	Recommendation	Implementing a central IHT coordination point	2, 13
	Recommendation	Improving transparency for German EMS workers regarding the treatments at certain Dutch hospitals	1
	Recommendation	Improving information exchange regarding available cross-border resources (special resources; GPS locations)	7

#### Communication Between Dispatch Centers

Concerning the communication between the dispatch centers in general, certain multi-language documents from the EMRIC partnership exist, which allow resource requests from neighboring dispatch centers. One expert considered the documents as not user-friendly and too time-consuming. Most dispatchers just directly call the other dispatch centers to ask for help (ID 7).

After requesting an ambulance from The Netherlands, the German dispatch center does not get any updates on the status of the mission until the dispatch center in Maastricht informs them where the patient has been transported to. This is very delayed and insufficient information exchange (ID 7). Consequently, the German dispatch center cannot answer the questions of second callers on the status of an alerted ambulance (e.g., the estimated time of arrival or the global positioning system (GPS) location) (ID 7). A summary of the findings is gathered in Table 8.

**Table 8:** Examples of lessons learned and recommendations concerning communication and requests for help between dispatch centers (based on encountered challenges before and during the first three COVID-19 waves).

**Area**	**Lessons learnt**	**Recommendations**	**Participants (ID)**
Communication and request for help between dispatch centers	Lesson learnt	Cross-border request of resources possible *via* multi-lingual EMRIC documents; documents are time-consuming and complex therefore dispatchers prefer direct communication (DE, NL, BE)	7
	Lesson learnt	Delayed and ineffective information exchange after request for help from NL was issued by DE; DE cannot update secondary callers on ambulance status (incl. time of arrival)	7

#### Technical Issues/Interoperability

A digital information screen exists at the dispatch center Heinsberg (DE), where cross-border information (e.g., on large fire incidents) appears for every dispatcher to see; but there is no connection between this screen and the German dispatching software, thus the information needs to be typed into their system manually (ID 7).

Connecting the dispatch systems in the EMR could improve the visibility of vehicles' locations once dispatched and could allow for translation opportunities, as some terms can be difficult and may lead to confusion (ID 7).

One expert explains a technical issue which caused a delay in alerting the emergency physician on German ground by a German ambulance team close to the Dutch border (ID 6). This emphasizes the importance of securing a mobile phone connection with the team's own dispatch center (ID 6).

“*But then the problem came to light that they did not have a good connection to the dispatch center via the work cell phone and the colleague then had to call with the private cell phone afterward because [the call] always ended up in The Netherlands. That must no longer be the case today. This is a technical problem, I think, that can be solved. […] That you can really reach […] the dispatch center that guides you and sends you in with support. And if you have a Dutch person on your ear in such a situation, who may speak German or English but then you have a resuscitation, […]. That's stupid then. […] but that can of course also be optimized by properly setting the cell phones that we have on the vehicles and routing them correctly. […]” (ID 6)*.

This was also explained by another expert, as the radio signal does not remain intact near the Dutch border (ID 7). In addition, communication between the dispatch center in Maastricht and the German helicopter remains difficult (ID 8). Even though a working group was set up for cross-border radio communication in the EMR, this is still an important and problematic issue for two experts (ID 7, 14). A summary of the findings is gathered in Table 9.

**Table 9:** Examples of lessons learned and recommendations for technical issues and interoperability (based on encountered challenges before and during the first three COVID-19 waves).

**Area**	**Lessons learnt**	**Recommendations**	**Participants (ID)**
Technical issues/Interoperability	Lesson learnt	Dispatch center Heinsberg: Major cross-border incidences are presented on a large information screen but the information is not directly connected to the dispatch system and can only be entered manually into the German dispatch system	7
	Recommendation	Dispatch systems in the EMR should be connected to improve visibility and communication (incl. resource locations and status, automatic translator)	7
	Lesson learnt	Radio and telephone compatibility is crucial but currently largely lacking [incl. (cellphone) service problems in border regions] (between foreign EMS and dispatch centers)	3, 6, 7, 14
	Lesson learnt	Radio communication between NL and the German helicopter remains difficult; A Dutch radio is currently built into the helicopter to solve communication issues	3, 8

## Discussion

The aim of this study was to demonstrate the impacts of and the lessons learned from the first three COVID-19 waves on the cross-border collaboration in EMS and IHT in the EMR. The interviewed experts were from various backgrounds, including those working at the political and crisis management level, medical directors (EMS), dispatch center managers, physicians, and dispatch center staff, firefighters, and EMS practitioners. The majority of those interviewed essentially agreed that cross-border collaboration in the EMR is indispensable. The present study highlights that the logistical challenges and disruptions widely experienced due to the ongoing pandemic call for improved and more robust collaboration across borders. While the capabilities that lie within the EMR have been highlighted before (9, 10, 36), the impacts of the pandemic make these even more apparent. A huge potential lies in exchanging best practices, organizing collaborative exercises, working together, and supporting each other with resources when needed (7). Still, this study reveals that, generally, prior to and during the pandemic, several issues and problems remain which hinder or handicap cross-border collaboration. This has also been identified by the Interreg-funded project PANDEMRIC, which analyses the cross-border cooperation within the EMR and presented the first results in two symposia in 2021 (37). While a common cross-border or even European approach in handling EMS and IHT missions or even general healthcare is sought, it does bring up challenges at the national policy-making level (3, 38). Fragmentation in the countries' national approaches to tackling the pandemic became clear and hindered the cross-border collaboration in the EMR to the extent of a temporary near-standstill. This was a result of the national measures and processes causing uncertainty and hindering widely established habits with the onset of the pandemic. In addition, this study identified several recommendations and lessons learned regarding the general collaboration, formal Euregional agreements, process changes, availability of cross-border resources, communication between dispatch centers, and the interoperability of technical systems. Knowledge regarding already existing official cross-border agreements could be improved at a larger scale while working toward even more legalized standardized cross-border procedures (3, 9, 36, 38). With the experience of the COVID-19 crisis, a new fresh look at the relevance and importance of EU Cross-Border Mechanisms as suggested during the EU presidency of Luxembourg in 2015 might reveal a better understanding of the need for a comprehensive and targeted set of tools that provide a sound legal basis for deviating from conflicting national regulations in the interest of necessary cross-border arrangements (39).

At a national level, cross-border concerns were not often considered in COVID-19 related policymaking and the implementation of national measures. Experts also reported that regional crisis management teams did not discuss the topic in depth. However, data on COVID-19 cases and bed capacity was exchanged during the course of the pandemic in the EMR. While two of the main characteristics of EMR are accessibility and easy transfer (10), the fragmentation among national measures such as border closures did not only cause national borders to become more visible again but also brought up uncertainty regarding operational processes. Other studies have also identified the fragmentation in the EU regarding nationally implemented COVID-19 measures (4, 5, 9). Previous studies highlighted the need for better European structures and a better delegation of tasks (3–5, 38, 40). The purchase and distribution of certain equipment call for joint procurement and standardized processes (4, 5, 40). This becomes especially apparent in the studied cross-border region, where countries often face similar obstacles or could help each other even better if standardized structures or agreements were in place and considered nationally (3). While a recent report by the project euPrevent COVID-19 states that national measures were regularly communicated across the border *via* one main contact point (6), this information seemed to have lacked at the operational level. Thus, a clear distinction must be made here between political decision-making and information reaching the operational workers.

Almost all challenges in this study can be attributed to the lack of standardized cross-border information exchange among the relevant stakeholders. Certain measures implemented by the EMRIC partnership have facilitated information exchange to some extent. In the EMR, additionally, a Euregional dashboard has been created showing specific COVID-19 data in the region (41). At the operational level and especially from the perspective of the dispatch centers, the implementation of real-time data exchange (on EMS and hospitals resources) and interoperable systems across the border would be a major improvement to request and offer help. Other EMS literature highlights the importance of continuous data collection and exchange between stakeholders involved at an operational level (3, 9). The main issues related to this concern the allocation of resources, lack of and dispatch of resources, as well as demand forecasting and the scheduling of IHT (2). In cross-border missions, these factors are even further highlighted. This study demonstrates that once a foreign resource has crossed the border, information exchange often completely stops, causing communication and demand-planning issues. Thus, systems and processes related to information exchange at the operational level need to be harmonized.

The well-established collaboration supported by the EMRIC group helped to facilitate structured and regular communication, also during the pandemic, which has also been identified in previous findings (6, 9). However, overall, the crisis also highlighted the limitations of the collaboration that is mainly based on mutual understanding and is missing any robust legal foundation or clear political mandate. The trust and mutual understanding developed during the long-term collaboration in this border region helped to find informal solutions and alternative practices to decisions on a national level that interfered with the established cross-border operational processes in order to maintain a level of continuity even during the crisis. But the COVID-19 “stress-test” of existing cross-border collaboration clearly shows the need for new more formal and legally binding arrangements to improve the resilience of public safety in the EMR in crises. Further, our study revealed major differences in the views of experts at the managerial/political level and those at the operational level, especially regarding the feasibility of measures implemented before and during the crisis. This also indicates some weaknesses in the governance structure of the cross-border collaboration on public safety in the region. The perceived detachment of leadership from operations can also be attributed to the informality of the governance structure and the absence of formal procedures and processes that guarantee transparency and participation.

Throughout the management of hospitalizations and ICU patients, cross-border collaboration remained intact at the beginning of the pandemic. Early findings support this, as various patients have been transported to other EU countries in the early phases of the pandemic (42). Learning from each other's experiences across the border has been identified as helpful in adapting national decision-making. Belgium for ex., conducted a study to learn from other EU countries by investigating surge capacity strategies in a selected number of countries, including Germany and The Netherlands (43). However, once national organizational structures were implemented to manage the IHT of patients with COVID-19, they overruled cross-border habits for patient transfers within the EMR, causing a clear gap between political decision-making and operational practicality. Thus, benefits such as the proximity of facilities were neglected during crucial decision-making processes. The need for a supra-regional/international system for patient allocation during COVID-19 based on shortages of ICU beds and other resources has been highlighted in previous findings (9, 40, 44) and underlines this identified issue in this study. Another study highlights the need for common infrastructures to monitor cross-border resources in the EU. The authors state that the transfer of patients across national borders was the first step toward improved allocation and solidarity (40). However, a high variety of available infrastructures to report hospital capacity was identified, especially across the EU. Some systems allow for real-time data collection and others did not have appropriate data infrastructures to allow the daily recording of numbers (9, 40).

## Recommendations

Generally, the following recommendations can be made for cross-border collaboration in EMS and IHT in the EMR:

- The cooperation would benefit from and be further strengthened by formal and legally binding Euregional agreements on operational processes to organize cross-border collaboration in EMS and IHT.
- Knowledge about official Euregional agreements and operational processes needs to be prioritized at the policy and operational level and governance structures need to improve participation and transparency.
- Considering and facilitating respective cross-border collaboration at the national policy-making level is essential to enhance cooperation among the neighboring countries.
- Establishing information exchange and technical system compatibility at the Euregional level to facilitate cross-border collaboration among the involved actors.
- Involving representatives of neighboring services in regional crisis management discussions to encourage information exchange and adaption of processes and enhance cooperation among the three countries.
- Identification and application of best practices and synergies at the Euregional level to encourage cross-border collaboration among the involved actors.

## Limitations

The study and its results have several limitations: Establishing contacts to experts was extremely difficult during the pandemic as all experts and potential interviewees were involved and responsible as front-line staff of the EMS services and hospitals prioritizing their medical and managerial duties. Therefore, the number of respondents is not as comprehensive as desired and originally intended. In addition, due to the busy schedule of the respondents, it turned out to be not feasible to have complete coverage for each of the different relevant stakeholder categories (medical director EMS, hospital/emergency physician, political level/crisis management team, ambulance/firefighting service, dispatch center) in each region. Due to the pandemic situation, the interviews were performed online (or by telephone if technical difficulties occurred).

The abovementioned challenges may limit the generalization of our findings. Further, the study only includes the results of the first three waves of the COVID-19 pandemic.

## Conclusion

As emergency situations or public health threats do not stop at borders, the cross-border collaboration of regional public safety services is highly important and in many European cross-border regions embedded in the European identity of the regional population. Falling back on stringent national reorganization and policy decision-making when trying to manage major crises like the pandemic should not disenable or hamper cross-border collaboration. The resilience of public safety in cross-border regions depends on sound and reliable regulations and legal tools that allow for the necessary cross-border support. To be effective, it is imperative that healthcare professionals at all levels (from operational up to political) are well-informed about cross-border resources and competencies to strengthen the cooperation among the EU Member States. Governance structures and decision-making for cross-border collaboration need to be based on robust legal instruments and the principles of transparency and participation. Identifying which ambulance could be fastest at the emergency scene or which suitable hospital is closest for a patient needing immediate care independent of national borders and organizational differences is an achievement of European collaboration. This pandemic clearly calls for the improvement of instruments and the political will and understanding to further strengthen cross-border collaboration and make the best use of scarce resources based on solidarity and mutual understanding.

## Data Availability Statement

The qualitative datasets presented in this article are not readily available because of privacy and data protection rules. Requests to access the datasets should be directed to Anja Sommer, ansommer@ukaachen.de; Cassandra Rehbock, crehbock@ukaachen.de. Any additional data used is available in the article or in the Supplementary Material.

## Ethics Statement

The study received ethical approval by the Ethics Committee of the Medical Faculty of RWTH Aachen, Germany (registration numbers: EK 390/20, CTC-A 20-417). The participants provided their written informed consent to participate in this study.

## Author Contributions

AS, CR, EP, MG, SB, and TK conceptualized and designed the study. AS and CR were the coordinating investigators and analyzed the findings. CV provided background information and illustrations. AS, CR, MG, SC, and SD conducted the interviews. AS, CB, CR, and SD coded the interviews. HS, SB, and TK supervised the work. AS drafted the manuscript and revised it. CR critically revised the manuscript. All authors revised and approved the manuscript.

## Funding

The study has been initiated and supported by the International Knowledge and Information Centre in public safety (IKIC) and received partial funding from Interreg EMR funds, funding number EMR77, website: https://www.ikic-publicsafety.eu/.

## Conflict of Interest

The authors declare that the research was conducted in the absence of any commercial or financial relationships that could be construed as a potential conflict of interest.

## Publisher's Note

All claims expressed in this article are solely those of the authors and do not necessarily represent those of their affiliated organizations, or those of the publisher, the editors and the reviewers. Any product that may be evaluated in this article, or claim that may be made by its manufacturer, is not guaranteed or endorsed by the publisher.

## Abbreviations

EMR: Euregio Meuse-Rhine
EMS: Emergency Medical Service(s)
IHT: Interhospital transport(s)
MICU: Mobile Intensive Care Unit.
