# Supplementary material for: Impacts and Lessons Learned of the First Three COVID-19 Waves on Cross-Border Collaboration in the Field of Emergency Medical Services and Interhospital Transports in the Euregio-Meuse-Rhine: A Qualitative Review of Expert Opinions
Source: Front Public Health. 2022 Mar 15;10:841013. doi: 10.3389/fpubh.2022.841013 (PMC8965022; doi:10.3389/fpubh.2022.841013)
Supplement: Supplementary file 1 [file Data_Sheet_1.pdf]

Supplementary Material

1 Interview guide

You have just been informed about the nature and goal of the interview - Do we have your consent to conduct and record this interview?

General information

- profession
- place of work
- operational area / deployment area

Specific information

1. How is patient allocation usually performed?
    - Is there a specific platform which is used for bed resource planning?
2. How is cross-border care usually organised in terms of EMS missions and IHT?
3. Are you aware of any cross-border agreements or habits / processes in your region?
4. Which measures were and are taken specifically for the Covid-19 pandemic? How and when were they communicated? (ex. hygienic measures, safety measures, operational changes, border-closing, allocation of bed resources)
    - national
    - regional
    - cross-border
5. Were there specific measures concerning cross-border collaboration discussed in the regional crisis management teams?
    - Was there any information communicated concerning cross-border care during the pandemic?
6. What are your experiences for cross-border cooperation during this time? What is the process and was it different to before the pandemic? (e.g. less missions, less transports, difference in IHT organisation, problems / issues for example when crossing the border or other)
    - Communication / Organisation

- differences general patients / Covid-19 patients (especially refer to bed resource planning and IHT)

- differences 1st and 2nd wave of pandemic

- refer to differences between Germany, Netherlands & Belgium (e.g. was there a collaboration with all?)

7. Do you know whether Euregional agreements or habits were still applicable during the first wave of the pandemic?

    - How did national / regional defined strategies (= agreements, habits, procedures or similar) conflict with Euregional agreements for EMS missions and / or IHT?

    - Which consequences could be seen (were Euregional agreements undermined by national defined strategies?)?

8. Do you think we were generally prepared for a situation like this?

    - can you state some examples please

9. Which lessons were learned from the first wave and which measures were taken to improve collaboration in the second wave?

10. What would you plan / do differently in another pandemic?

11. Do you have any other points of interest which we did not discuss / address yet?

2 Figure on national measures during COVID-19 pandemic

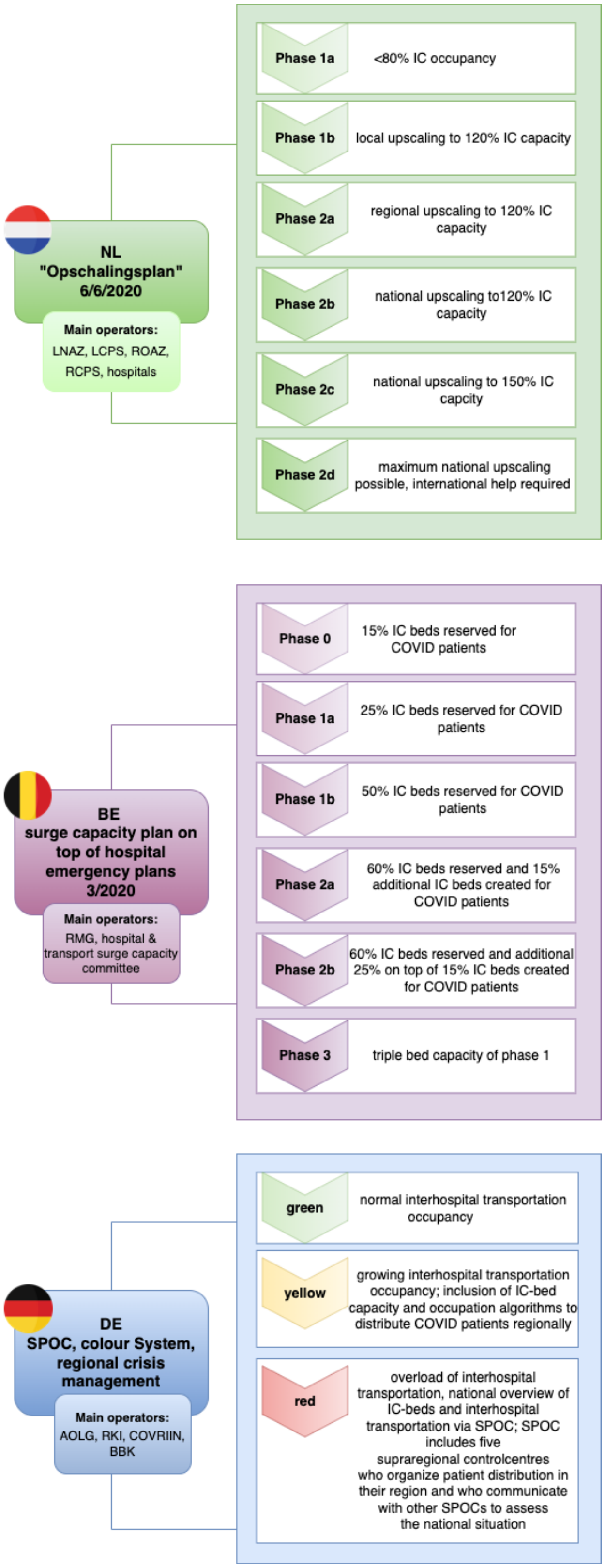

Figure 1. National measures in the Netherlands, Belgium and Germany during the COVID-19 pandemic (1–3)

References for Figure 1

1. Landelijk Netwerk Acute Zorg. Opschalingsplan COVID-19. (2020)

2. Gesundheitsministerkonferenz. Kleeblattkonzept für einen länderübergreifenden Patiententransport bei einem worst-case COVID-19-Szenario. (2020).

3. van de Voorde C, Lefèvre M, Mistiaen P, Detollenaere J, Kohn L, van den Heede K. Assessing the management of hospital surge capacity in the first wave of the COVID-19 pandemic in Belgium. Brussels: Belgian Health Care Knowledge Centre (KCE) (2020). www.kce.fgov.be

3 Cross-border missions

Cross-border missions: the following 6 figures present the number of cross-border missions collected by the EMRIC partners in 2019 and 2020.

These figures show which emergency services from the title region conducted cross-border missions in the two countries presented in the colored bars.

Each green box highlights the respective total number of missions (= medical total).

This data was provided by the Fire department Aachen as part of EMRIC.

B = Belgium, D = Germany, NL = The Netherlands

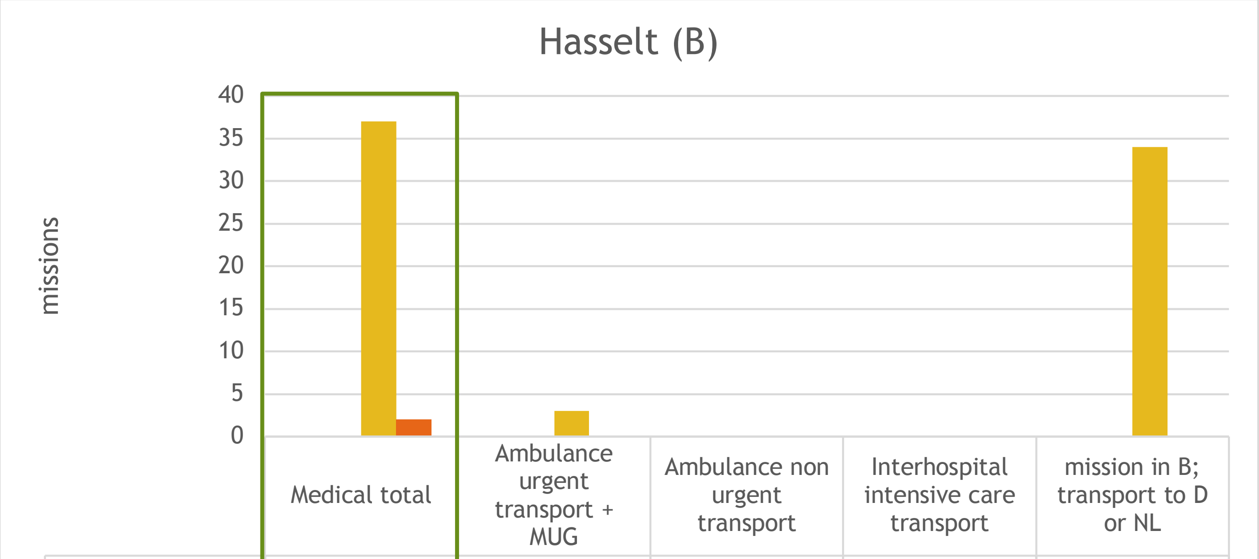

	Medical total	Ambulance urgent transport + MUG	Ambulance non urgent transport	Interhospital intensive care transport	mission in B; transport to D or NL
Germany 2019	0	0	0	0	0
Germany 2020	0	0	0	0	0
Netherlands 2019	37	3	0	0	34
Netherlands 2020	2	0	0	0	0

Figure 2. Hasselt (B)

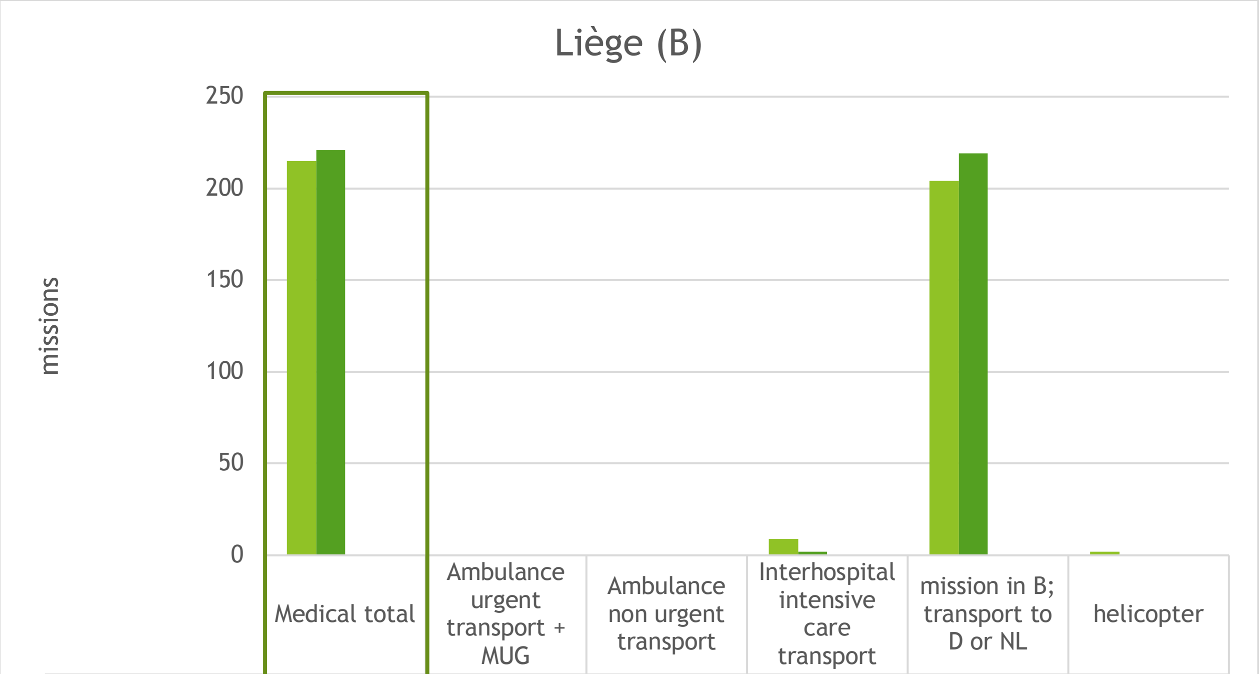

	Medical total	Ambulance urgent transport + MUG	Ambulance non urgent transport	Interhospital intensive care transport	mission in B; transport to D or NL	helicopter
Germany 2019	215	0	0	9	204	2
Germany 2020	221	0	0	2	219	0
Netherlands 2019	0	0	0	0	0	0
Netherlands 2020	0	0	0	0	0	0

Figure 3. Liège (B)

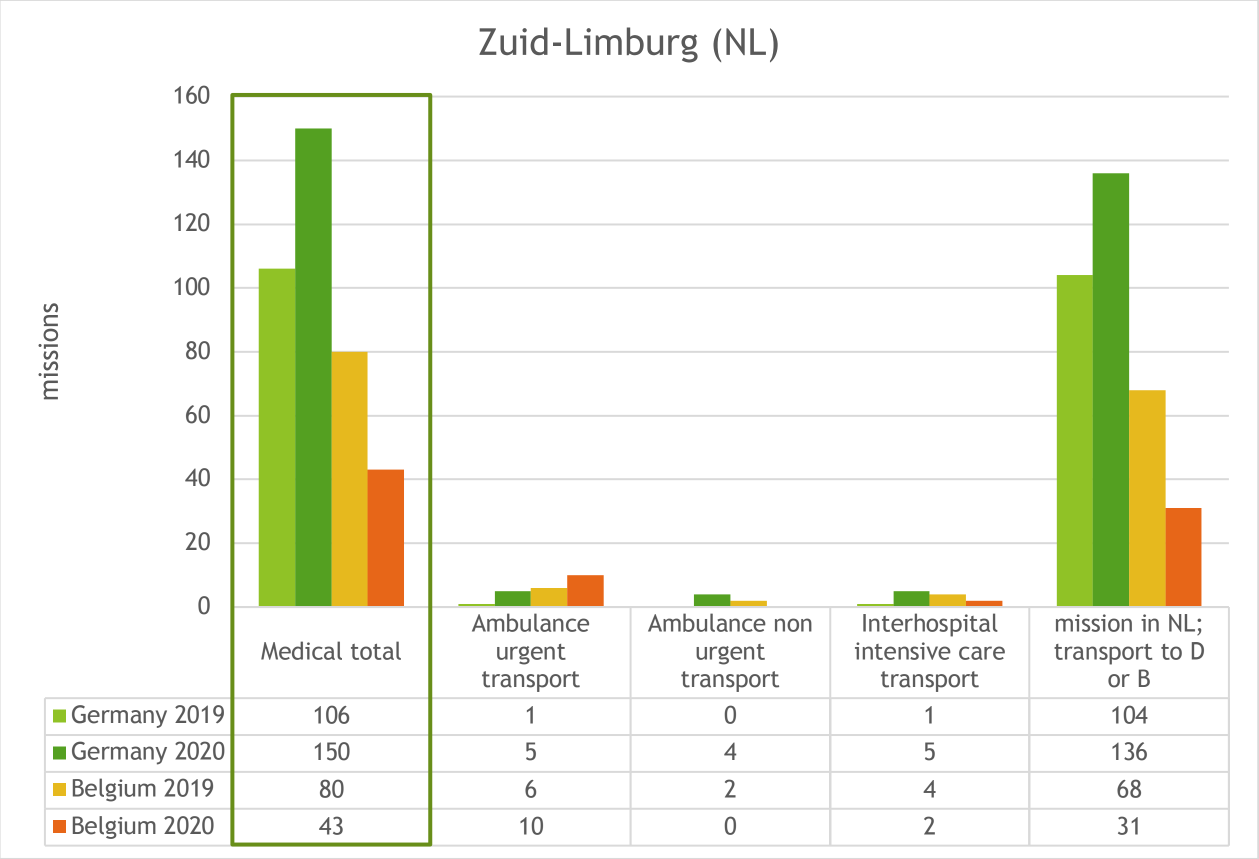

	Medical total	Ambulance urgent transport	Ambulance non urgent transport	Interhospital intensive care transport	mission in NL; transport to D or B
Germany 2019	106	1	0	1	104
Germany 2020	150	5	4	5	136
Belgium 2019	80	6	2	4	68
Belgium 2020	43	10	0	2	31

Figure 4. Zuid-Limburg / South Limburg (NL)

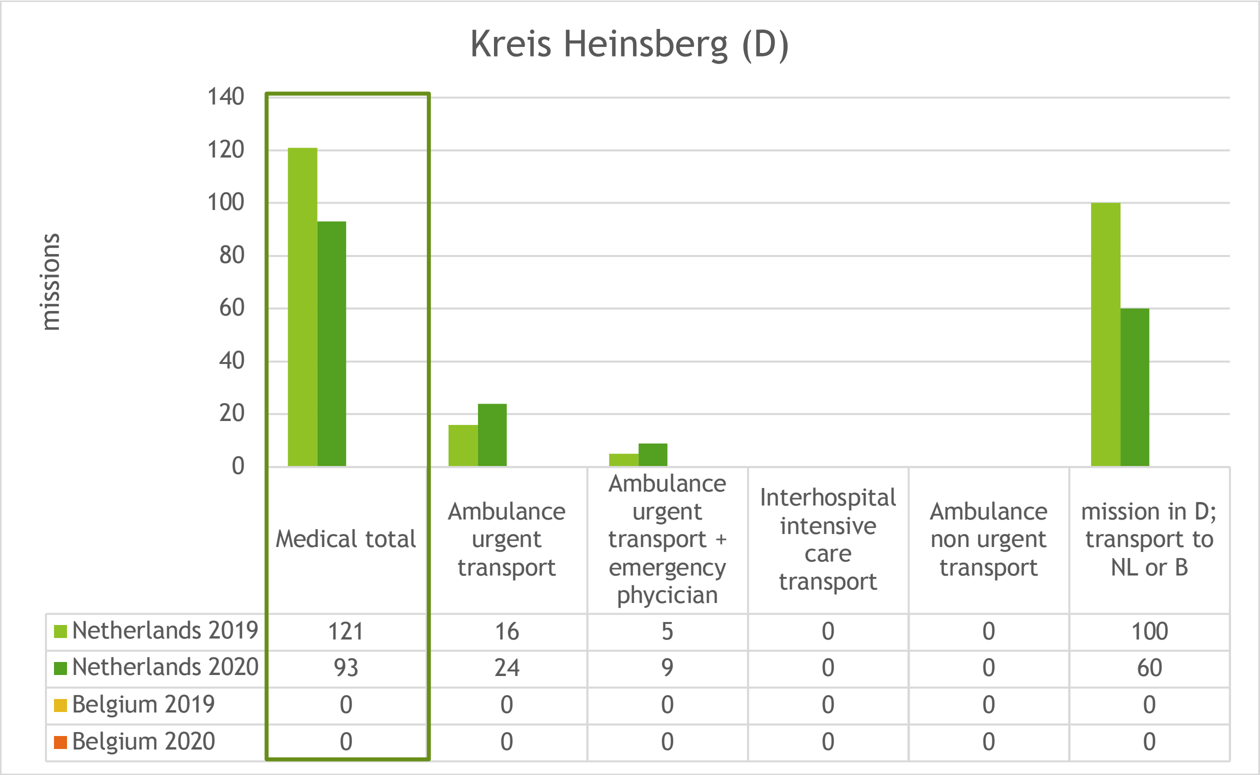

Figure 5. Kreis Heinsberg / District of Heinsberg (D)

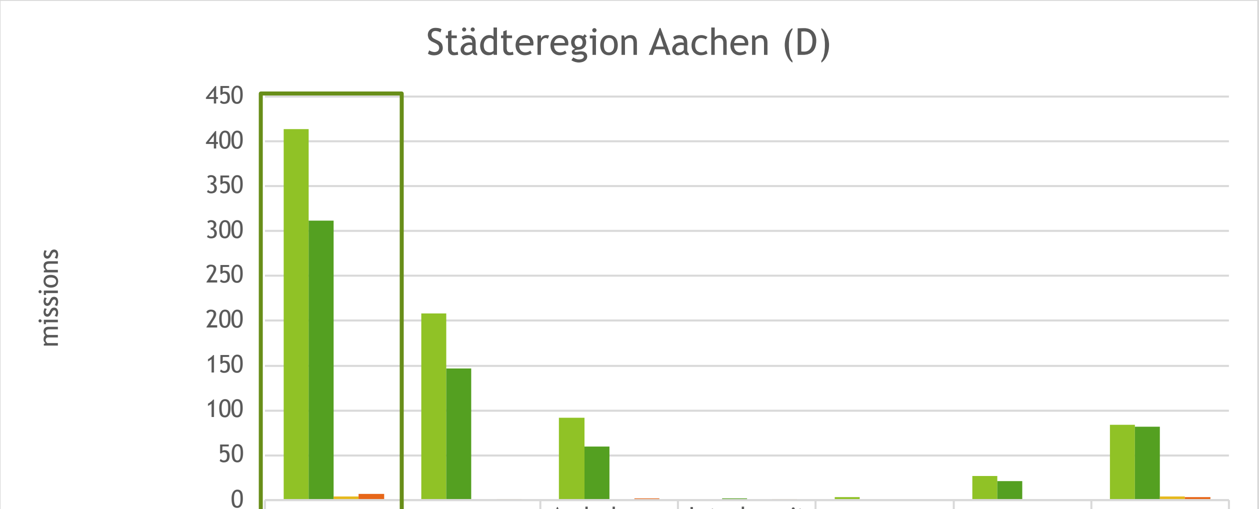

	Medical total	Ambulance urgent transport	Ambulance urgent transport + emergency phycician	Interhospital intensive care transport	Ambulance non urgent transport	mission in D; transport to NL or B	helicopter
Netherlands 2019	414	208	92	0	3	27	84
Netherlands 2020	312	147	60	2	0	21	82
Belgium 2019	4	0	0	0	0	0	4
Belgium 2020	7	1	2	1	0	0	3

Figure 6. Städteregion Aachen / City region of Aachen (D)

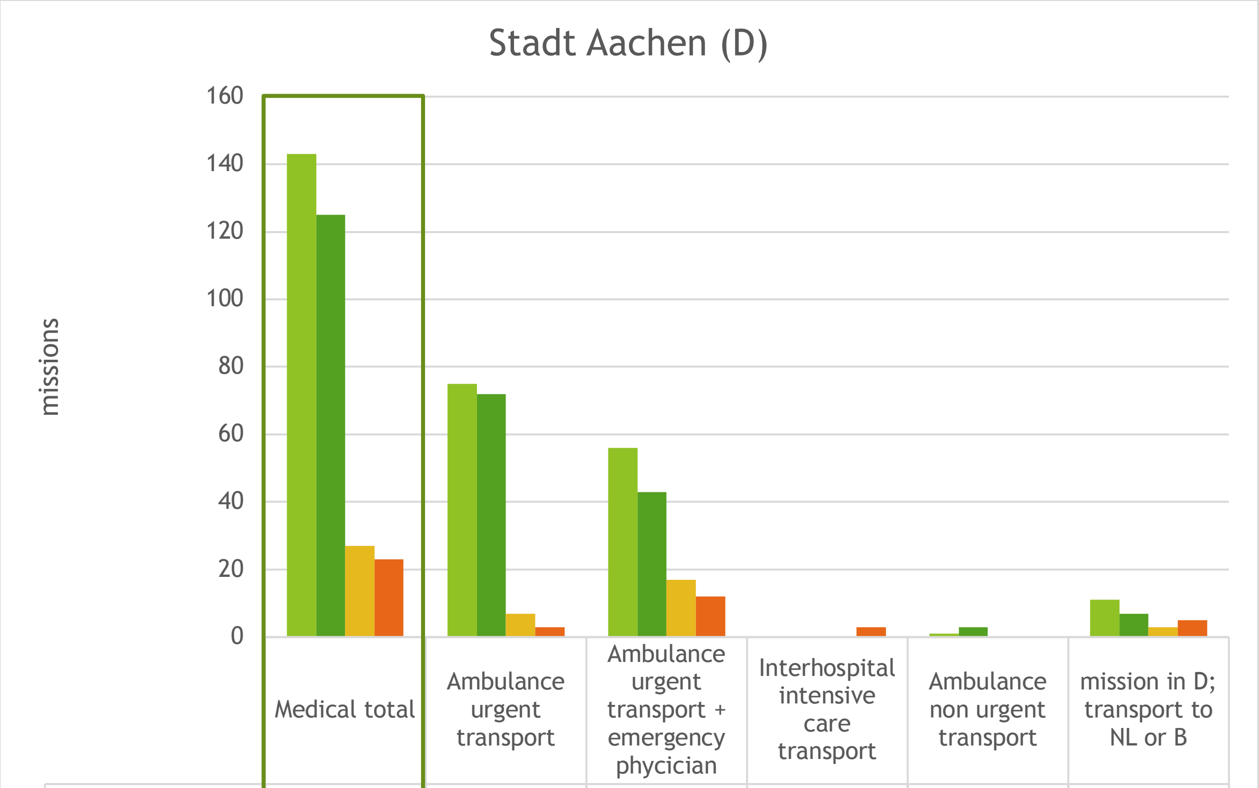

	Medical total	Ambulance urgent transport	Ambulance urgent transport + emergency phycician	Interhospital intensive care transport	Ambulance non urgent transport	mission in D; transport to NL or B
Netherlands 2019	143	75	56	0	1	11
Netherlands 2020	125	72	43	0	3	7
Belgium 2019	27	7	17	0	0	3
Belgium 2020	23	3	12	3	0	5

Figure 7. Stadt Aachen / City of Aachen (D)
